# Length of stay as quality indicator in emergency departments: analysis of determinants in the German Emergency Department Data Registry (AKTIN registry)

**DOI:** 10.1007/s11739-021-02919-1

**Published:** 2022-01-06

**Authors:** Ronny Otto, Sabine Blaschke, Wiebke Schirrmeister, Susanne Drynda, Felix Walcher, Felix Greiner

**Affiliations:** 1 Department of Trauma Surgery, Otto Von Guericke University, Leipziger Str. 44, 39120 Magdeburg, Germany; 2 Emergency Department, University Medicine Göttingen, Göttingen, Germany

**Keywords:** Emergency department, Length of stay, Quality indicator, Registry

## Abstract

Several indicators reflect the quality of care within emergency departments (ED). The length of stay (LOS) of emergency patients represents one of the most important performance measures. Determinants of LOS have not yet been evaluated in large cohorts in Germany. This study analyzed the fixed and influenceable determinants of LOS by evaluating data from the German Emergency Department Data Registry (AKTIN registry). We performed a retrospective evaluation of all adult (age ≥ 18 years) ED patients enrolled in the AKTIN registry for the year 2019. Primary outcome was LOS for the whole cohort; secondary outcomes included LOS stratified by (1) patient-related, (2) organizational-related and (3) structure-related factors. Overall, 304,606 patients from 12 EDs were included. Average LOS for all patients was 3 h 28 min (95% CI 3 h 27 min–3 h 29 min). Regardless of other variables, patients admitted to hospital stayed 64 min longer than non-admitted patients. LOS increased with patients’ age, was shorter for walk-in patients compared to medical referral, and longer for non-trauma presenting complaints. Relevant differences were also found for acuity level, day of the week, and emergency care levels. We identified different factors influencing the duration of LOS in the ED. Total LOS was dependent on patient-related factors (age), disease-related factors (presentation complaint and triage level), and organizational factors (weekday and admitted/non-admitted status). These findings are important for the development of management strategies to optimize patient flow through the ED and thus to prevent overcrowding.

## Introduction

Complexity of emergency treatment as well as the decision of priority have a substantial impact on emergency department (ED) processes [1, 2]. The process within the ED usually starts with the admission of the emergency patient, followed by acuity assessment (triage), diagnosis, treatment initiation, and decisions on discharge or further inpatient treatment [3]. The periods between these procedures are defined as process times. They play an important role in the evaluation of the quality of care in EDs and are influenced by various factors, including patient flow, the severity of the condition, and category of disease or injury. Evaluation of these process times by using routinely collected data permits transparent characterization of most ED processes, and thus, allows for the development of improvement strategies in ED performance [4–6]. One of the most important indicators of ED performance is the length of stay (LOS), which represents a highly relevant quality indicator (QI) [7, 8]. Therefore, it is suitable as a tool for evaluating the process quality and performance of an ED [9, 10].

Modelling of patient flow in EDs by established methods such as event simulation has previously been described in numerous studies, providing essential approaches for the optimization of ED performance [11–13]. Overcrowding is one of the most common problems observed in EDs and is mainly caused by an excessively long LOS within the ED, leading to a disruption of all processes and a dramatic increase in risks of emergency treatment [14–16]. Therefore, it is important to evaluate all factors influencing the process times within the ED. These factors are classified into different categories: patient factors (demographic and health-related factors), organizational factors, and structural factors (emergency care levels).

In a systematic review by Hörster et al., 15 different process times, including repeatedly cited QIs, for EDs were described [17]. Using routine data taken from the Emergency Department Medical Record V2015.1 collected within the German Emergency Department Data Registry (AKTIN registry), six of the 15 process times could be calculated, namely, arrival up to initial clinical assessment, arrival to initial triage, arrival to initial treatment, time to computer tomography, LOS of admitted patients, and LOS of non-admitted patients [18].

Previous studies on determinants were usually monocentric, based on small cohorts and only examined selected factors [19, 20]. In order to identify factors that can be influenced, all possible determinants should be taken into account. In addition international studies are of limited value for analyzing the German health care system due to different, state specific organizational and structural characteristics of emergency care systems. In addition, no symptoms have been recorded in routine data sets so far. This study analyzed the LOS in the ED and its determinants by evaluating data sets in the AKTIN registry. Results were compared with international time targets that have previously been introduced in various countries to improve ED performance [21–24].

Methods

### Patients and study setting and data basis

In 2019, 16 German hospitals, with an average total patient volume of about 40,000 emergencies per month, participated in the AKTIN registry. They are located throughout Germany, include university as well as non-university hospital EDs and cover all three levels of emergency care according to the specifications of the Federal Joint Committee [25], the highest decision-making body of the joint self-government of physicians, dentists, hospitals and health insurance funds in Germany. These levels are basic emergency care, extended emergency care and comprehensive emergency care. This subdivision was introduced in 2018 with regard to remuneration and is based on type and number of specialist departments, staffing with specialists, capacity to care for intensive care patients, medical-technical equipment and structures and processes of the ED. The base module of the German Emergency Department Medical Record V2015.1, published by the German Interdisciplinary Association of Critical Care and Emergency Medicine (DIVI e. V.), defines items such as the demographic data, Canadian Emergency Department Information System (CEDIS) Presenting Complaint List [26], acuity assessment (triage) according to the Manchester Triage System (MTS), and the Emergency Severity Index (ESI) as well as vital parameters, date/time, and diagnoses coded by the German modification of the International Classification of Diseases and Related Health Problems, 10th revision (ICD-10-GM) at the end of the ED treatment [27]. In this study, LOS was defined as the interval between *t*0 and *t*1: *t*0 refers to the first timestamp recorded in the system (possible data items: time of administration, time of initial assessment, time of first physician contact), and *t*1 is the timestamp of patient disposition (i.e., discharge in case of outpatient treatment or transfer in case of hospital admission). The data were documented for all patients as part of the routine documentation with the respective hospital information system.

The anonymized data were retrieved via the AKTIN registry (Project-ID 2020-001), enabling data protection-compliant access to the data stored in local data warehouses. Individual patient consent is not feasible in the context of an emergency situation. Instead, there is a strict data protection concept for the AKTIN registry and technical and organizational measures ensure anonymity [18]. Data request was reviewed by the scientific committee (review board) with regard to scientific merit, feasibility, ethics, and data protection issues [18, 28, 29]. The data protection work group of the Technologies, Methods, and Infrastructure for Networked Medical Research approved the privacy policy of the AKTIN registry. The AKTIN project was approved by the ethics committee of Otto von Guericke University, Medical Faculty, Magdeburg (160/50–23.11.2015).

### Inclusion and exclusion criteria and plausibility check

All patients aged ≥ 18 years attending an ED participating in the AKTIN registry during the study period in 2019 were included in this study. Only complete datasets were used for statistical analyses. Cases in which the discharge date was not documented, before t0 or the calculated LOS seemed implausible were excluded. We used a threshold of 24 h as the upper limit for a very high, but still realistic LOS [30]. All patients who died in the ED were also excluded to avoid measurement errors of the LOS by evaluating the data item "time to death" as the time of discharge from the ED (i.e., pick-up time after death).

### Statistical analyses

First, LOS was calculated for all patients. Additionally, LOS was stratified for different subgroups with respect to the parameters age, sex, referral, initial assessment, and disposition (Table 1). LOS in relation to the initial assessment was differentiated between the two triage systems MTS and ESI owing to different algorithms [31]. Additionally, LOS was compared with respect to specific "time targets" [21–24] (Table 2). For further analysis, patients were assigned to traumatological and non-traumatological care according to their complaint listed in CEDIS [32] (Table 3). Finally, LOS was analyzed in relation to the days of the week and the seasonal pattern.

**Table 1:** Patients’ characteristics and LOS in the ED

*n* = 304,606	LOS (mins)			
	*N* (%)	M ± SD	95% CI (M)	Median
Total LOS		207.9 ± 162.5	207.3–208.5	171.3
*Age (years), M ± SD*	54.9 ± 22.0			
18–40	96,999 (31.8%)	174.1 ± 144.1	173.2–175.0	140.9
41–60	75,893 (24.9%)	203.8 ± 159.5	202.7–205.0	167.2
61–70	39,057 (12.8%)	226.9 ± 169.6	225.2–228.5	190.0
71–80	45,712 (15.0%)	237.1 ± 172.5	235.5–238.6	200.0
> 81	46,945 (15.4%)	240.1 ± 172.8	238.5–241.7	204.4
*Gender*				
Male	155,316 (51.0%)	203.8 ± 161.1	203.0–204.6	167.0
Female	149,123 (48.9%)	212.2 ± 163.9	211.3–213.0	175.9
Diverse/no information	167 (0.05%)	163.7 ± 153.9	140.1–187.2	124.8
Referral				
Walk-in patients	125,166 (46.0%)	176.0 ± 143.1	175.2–176.8	143.2
Emergency medical service	87,114 (32.0%)	235.7 ± 180.9	234.5–236.9	193.0
General practitioner (GP)	43,086 (15.8%)	239.3 ± 150.2	237.9–240.7	211.2
GP-based emergency care/practice	11,224 (4.1%)	225.9 ± 194.0	222.4–229.5	178.0
Referral from other clinics	5654 (2.1%)	225.1 ± 191.3	220.1–230.0	174.0
Total	272,244 (100%)	–		–
No information	32,362 (10.6%)			
*Acuity assessment (triage)*				
Red—immediate	4372 (1.6%)	^a^		–
Orange—very urgent	33,310 (12.0%)	–		–
Yellow—urgent	106,073 (38.3%)	–		–
Green—standard	123,593 (44.6%)	–		–
Blue—non-urgent	9803 (3.5%)	–		–
Total	277,151 (100%)	–		–
Without documented acuity assessment	27,455 (9.0%)	–		–
*Disposition*				
Discharge (non-admitted)	150,666 (57.1%)	183.5 ± 140.6	183.2–184.6	152.0
Hospital-admission (admitted)	98,592 (37.4%)	247.4 ± 185.0	246.2–248.5	204.0
Discharge against medical advice (non-admitted)	5292 (2.0%)	230.2 ± 132.2	226.6–233.7	205.9
No medical contact (non-admitted)	3392 (1.3%)	125.8 ± 176.8	119.8–131.7	70.2
Treatment aborted by patient (non-admitted)	2755 (1.0%)	196.3 ± 163.1	190.2–202.3	158.5
Transfer to other clinics	2021 (0.8%)	275.6 ± 222.4	265.9–285.3	216.8
Other discharge (non-admitted)	990 (0.4%)	153.0 ± 180.8	141.8–164.3	100.4
Total	263,708 (100%)			
Non-admitted	163,095 (62.3%)	183.9 ± 142.4	183.2–184.6	152.1
Admitted	98,592 (37.7%)	247.4 ± 185.0	246.2–248.5	204.0
Disposition not documented	40,898 (13.4%)	-		-

M, mean; SD, standard deviation; LOS, length of stay; 95% CI, 95% confidence interval of mean; ED, emergency department; MTS, Manchester Triage System; ESI, Emergency Severity Index

^a^The LOS for acuity assessment was considered separately for MTS and ESI in Table 4

**Table 2:** Time targets

Time targets	Country	AKTIN registry data			
		*N* (%)	LOS (mins)	LOS ADM	LOS non-ADM
			M ± SD (95% CI)	M ± SD 95% CI (% of *N*)	M ± SD 95% CI (% of *N*)
4 h	Australia (80%), England (98%), Canada (90% non-admitted)	211,997 (69.6%)	129.6 ± 59.9 (129.4–129.9)	145.3 ± 55.7 (144.8–145.7) (28.3%)	123.3 ± 60.0 (123.0–123.7) (58.0%)
6 h	New Zealand (95%)	268,582 (88.2%)	163.8 ± 86.3 (163.5–164.2)	184.9 ± 82.6 (184.4–185.5) (30.6%)	152.8 ± 85.1 (152.4–153.2) (55.5%)
8 h	Canada (90% admitted)	289,318 (95.0%)	181.5 ± 105.0 (181.1–181.8)	207.1 ± 103.8 (206.1–207.8) (31.5%)	166.6 ± 101.3 (166.1–167.1) (54.5%)

ADM, admitted; non-ADM, non-admitted; M, mean; SD, standard deviation; 95% CI, 95% confidence interval of mean; LOS, length of stay; AKTIN, the German Emergency Department Data Registry

**Table 3:** LOS (mins) in relation to presenting complaints, summarised for trauma and non-trauma, and the top 10

CEDIS code	LOS (min)	ADM	Non-ADM	ADM vs. non-ADM
*N* = 232,624 (70,798 (23.2%) missing data)	M ± SD (95% CI)	M (%)^a^ (95% CI)^a^	M (%)^a^ (95% CI)^a^	*p* (*r*)
Trauma (*n* = 50,208, 21.6%)	149.8 ± 121.8 (148.8–150.9)	219.9 (19.8) (216.2–223.6)	135.9 (80.2) (134.9–137.0)	< 0.001 (0.270**)
Non-trauma (*n* = 182,416, 78.4%)	223.5 ± 173.2 (222.7–224.3)	257.8 (44.6) (256.5–259.2)	199.7 (55.4) (198.7–200.7)	< 0.001 (0.192**)
251—abdominal pain (*n* = 19,795)	250.4 ± 175.9 (247.9–252.8)	272.1 (45.3) (268.2–276.0)	232.8 (45.4) (229.8–236.1)	< 0.001 (0.119**)
556—upper extremity injury (*n* = 13,077)	143.3 ± 105.6 (141.5–145.1)	215.1 (11.9) (207.4–222.9)	134.9 (75.5) (133.1–136.7)	< 0.001 (0.245**)
557—lower extremity injury (*n* = 12,544)	150.0 ± 105.8 (148.2–151.9)	211.2 (19.1) (205.8–216.6)	135.7 (64.0) (133.8–137.6)	< 0.001 (0.315***)
555—lower extremity pain (*n* = 11,542)	166.0 ± 109.5 (164.0–168.0)	214.5 (20.0) (209.4–219.6)	154.0 (73.3) (151.9–156.2)	< 0.001 (0.243**)
554—upper extremity pain (*n* = 10,586)	158.8 ± 110.1 (156.7–160.9)	216.4 (14.0) (209.1–223.6)	150.7 (79.4) (148.6–152.9)	< 0.001 (0.207**)
651—shortness of breath (*n* = 9,796)	246.9 ± 178.8 (243.4–250.4)	256.6 (62.6) (251.9–261.2)	243.9 (23.1) (237.4–250.5)	0.326 (0.010*)
003—chest pain/cardiac features (*n* = 9,692)	271.4 ± 204.4 (267.3–275.4)	296.3 (43.4) (289.3–303.3)	277.7(38.2) (272.1–283.2)	0.158 (0.015*)
551—back pain (*n* = 9,138)	197.2 ± 134.3 (194.4–199.9)	231.7 (29.2) (225.8–237.6)	183.7 (65.6) (180.7–186.7)	< 0.001 (0.158**)
409—extremity weakness/symptoms of CVA (*n* = 8,290)	237.1 ± 177.8 (233.3–240.9)	226.3 (73.9) (222.0–230.6)	265.6 (17.1) (256.0–275.2)	< 0.001 (0.116**)
704—laceration/puncture (*n* = 7,468)	128.9 ± 107.4 (126.4–131.3)	206.0 (11.1) (194.9–217.0)	121.6 (81.4) (119.2–123.9)	< 0.001 (0.246**)

CEDIS, Canadian Emergency Department Information System; ADM, admitted; Non-ADM, non-admitted; M, mean; SD, standard deviation; 95% CI, 95% confidence interval of mean; LOS, length of stay; CVA, cerebrovascular accident

*No effect, **small effect, ***moderate effect

^a^Related to all cases with documentation of a presenting complaint

All calculations and graphics were created using the R statistical software [33]. The individual process times were presented descriptively as mean ± standard deviation (if not noted otherwise), additionally the 95% confidence interval of the mean is reported. In Table 1, the median was also shown due to a lack of normal distribution of the data. Comparisons between LOS for binary variables were performed with the Mann–Whitney *U* test. For variables with more categories, the Kruskal–Wallis test was used. Dunn's test was used as a post hoc test for the Kruskal–Wallis test. Differences were considered statistically significant at *p* < 0.05. The *p* value was adjusted using the Benjamini–Hochberg procedure. Due to the high number of cases, even small differences can be significant. For this reason, the effect sizes *η*^2^ (Kruskal–Wallis) and *r* (Mann–Whitney* U*) were also indicated. These were interpreted with: *η*^2^ 0.01 to < 0.06 (small effect), 0.06 to < 0.14 (moderate effect) and ≥ 0.14 (large effect) [34]. The following values were applied to *r*: 0.10 to < 0.3 (small effect), 0.30 to < 0.5 (moderate effect) and ≥ 0.5 (large effect) [35].

## Results

Twelve AKTIN EDs participated in the study, which included four extended emergency care providers (EECP), seven comprehensive emergency care providers (CECP), and one basic emergency care provider (BECP). The resulting dataset included 321,498 cases. Participating hospitals provided data sets from about 12,000 to 38,000 adult patients per clinic during the study period. Seven hospitals used MTS, and five hospitals used ESI as the triage system.

**Fig. 1:**
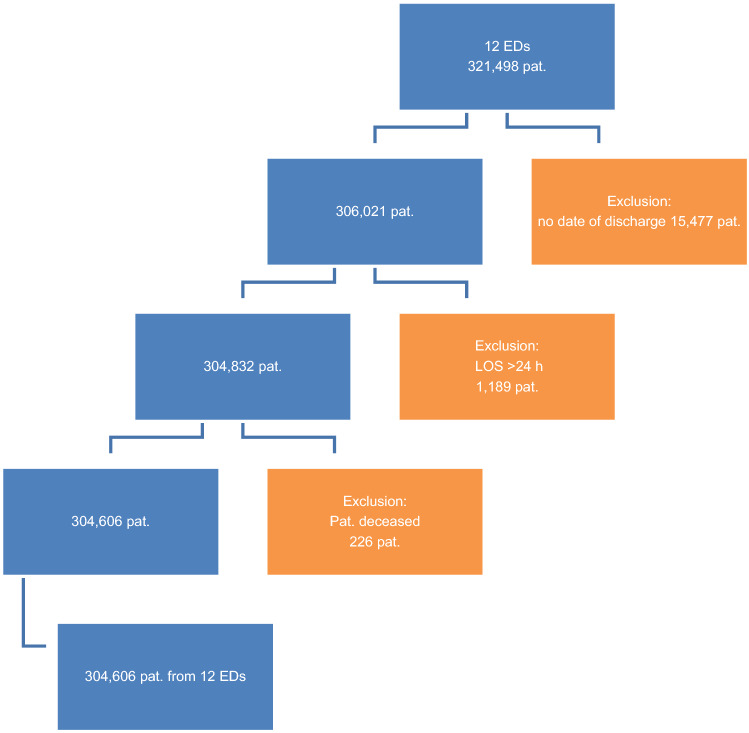
Evaluation of AKTIN registry data according to inclusion and exclusion criteria. ED, emergency department; pat., patients

A total of 100% of the admission dates were available. The following data sets were excluded: 15,477 (4.3%) data sets due to missing discharge dates; 1189 records (0.38%) with a calculated LOS of > 24 h; and 226 (0.07%) patients who died in the ED. After data set cleaning, 304,606 valid data sets were included in the analysis as outlined in Fig. 1**.**

### LOS

The mean LOS in the ED was 3 h 28 min (95% CI 3 h 27 min–3 h 29 min) with a minimum of < 1 min and a maximum of 24 h (Table 1). The minimum LOS resulted from patients leaving the ED without contact with a physician or being transferred to another ward immediately after arrival (e.g., delivery room). Patients admitted or transferred to another hospital mainly accounted for the maximum LOS.

Of all patients, 69.6% left the ED within four hours. Admitted patients had a 20 min higher LOS than non-admitted patients. Within 6 h, 88.2% of the patients were discharged, with an average LOS of 2 h 43 min (95% CI 2 h 43 min–2 h 44 min). 5% of all emergency patients left the ED after 8 h, their mean LOS was 11 h 48 min (95% CI 11 h 44 min–11 h 51 min), with 53.1% of admitted patients in this group (Table 2).

### LOS depending on patient-related factors

The analysis of demographic data (Table 1) revealed no gender differences in LOS in male and female ED patients (51.0% vs. 48.9%) with an average LOS of over 3 h. LOS increased significantly with respect to the age of ED patients. The mean age was 55 ± 22 years, with women being on average 2 years older than men (56 vs. 54 years). Patients aged between 18 and 40 years stayed in the ED for an average of 2 h 55 min (95% CI 2 h 53 min–2 h 55 min), whereas patients aged ≥ 80 years were in the ED for up to 4 h. However, in contrast to the age group of 18–40 years with only 15.4% of admitted patients, 52% of those over 80 years were admitted to the hospital.

Most ED patients were walk-in patients (46.0%) who stayed for the shortest time (2 h 56 min, 95% CI 2 h 55 min–2 h 57 min). Only 19.4% of the patients were admitted. For the other types of referrals (25.8% via emergency medical service and 15.8% via the general practitioner), LOS was over 3 h 45 min (Table 1).

### LOS as a function of disease-related factors

The ten most frequent presenting complaints according to the CEDIS codes comprised 36.7% of all cases attending EDs. As outlined in Table 3, patients presenting with abdominal pain, cardiac chest pain, and shortness of breath exhibited the highest LOS. Patients with injuries and pain in the upper and lower extremities were released from the ED on average up to 1 h 40 min earlier than patients with abdominal pain, chest pain, and shortness of breath. Patients with laceration/puncture exhibited the shortest LOS. In all categories except for shortness of breath and cardiac chest pain, LOS was significantly longer for admitted patients than for non-admitted patients.

Overall, the LOS of non-trauma patients in the ED was 1 h 14 min longer than that of trauma patients (Table 3). Approximately 44.6% of non-trauma patients and 19.8% of the trauma patients were admitted to the hospital. In the case of hospital admission, non-trauma patients stayed for approximately 40 min longer in the ED than trauma patients (3 h 39 min vs. 4 h 17 min, *p* < 0.001, *r* = 0.082). Similarly, in the case of discharge, non-trauma patients had 60 min longer LOS than trauma patients (2 h 15 min vs. 3 h 19 min, *p* < 0.001, *r* = 0.228).

### LOS in relation to the acuity assessment (triage system)

**Fig. 2:**
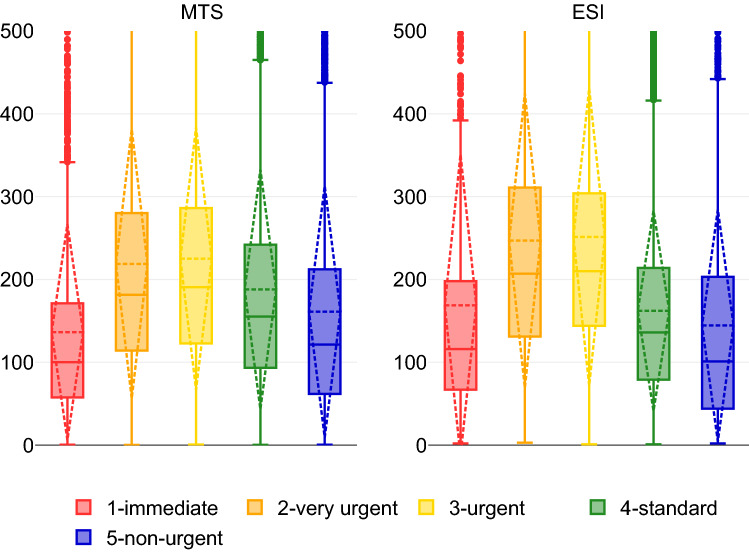
LOS (mins) for levels 1–5, MTS, and ESI for all cases. LOS, length of stay; MTS, Manchester Triage System; ESI, Emergency Severity Index

In hospitals using MTS as triage system (*n* = 7; LOS: 3 h 21 min (95% CI 3 h 20 min–3 h 22 min)), most patients were categorized as level 3—yellow (34.2%) and level 4—green (51.1%). Level 3 had an LOS of 3 h 45 min (95% CI 3 h 43 min–3 h 26 min), but fewer patients were admitted for treatment than patients in level 1—red or 2—orange. In case of admission, patients in level 1 had the shortest LOS of 2 h 3 min (95% CI 1 h 57 min–2 h 10 min), whereas patients in levels 3–5 stayed for an average of 4 h in the ED. Strong differences in LOS between triage levels (except for level 3 vs. level 5) were detected (*p* < 0.001). In non-admitted cases, patients in level 5 had the shortest LOS (2 h 24 min, 95% CI 2 h 21 min–2 h 28 min) and patients in level 2 had the longest LOS (4 h 10 min, 95% CI 4 h 5 min–4 h 16 min) in the ED. The LOS decreased from level 2–5 patients by approximately 2 h. Similarly, significant differences in the LOS between triage levels (except for level 1 vs. level 3) were also found in the non-admitted group (*p* < 0.001) (Table 4, Fig. 2).

**Table 4:** LOS in EDs using MTS (*n* = 7) or ESI (*n* = 5)

MTS (*n* = 7)	*N* (%)	LOS (mins)	ADM	ADM *p* < 0.001, *η*^2^ = 0.035*	Non-ADM	Non-ADM *p* < 0.001, *η*^2^ = 0.038*	ADM vs. Non-ADM
		M ± SD (95% CI)	%	M ± SD (95% CI)	%	M ± SD (95% CI)	*p* (*r*)
Red	2918 (1.59%)	136.4 ± 130.3 (131.6–141.1)	46.8	123.4 ± 116.3 (117.2 -129.6)	6.9	218.6 ± 192.3 (192.0–245.3)	< 0.001 (0.231**)
Orange	16,046 (8.7%)	218.8 ± 162.7 (216.3–221.3)	53.2	197.8 ± 158.3 (194.4–201.2)	22.6	250.7 ± 172.8 (245.1–256.3)	< 0.001 (0.199**)
Yellow	62,837 (34.2%)	225.2 ± 158.4 (223.9–226.4)	37.6	234.6 ± 167.3 (232.5–236.8)	43.8	206.6 ± 152.2 (204.8–208.4)	< 0.001 (0.105**)
Green	93,786 (51.1%)	187.9 ± 144.8 (187.0–188.9)	23.7	248.9 ± 170.5 (246.7–251.2)	67.9	166.8 ± 128.2 (165.8–167.8)	< 0.001 (0.279**)
Blue	7994 (4.4%)	161.1 ± 151.9 (157.8–164.4)	15.9	237.9 ± 183.9 (227.8–248.0)	74.6	144.6 ± 135.9 (141.1–148.0)	< 0.001 (0.259**)

ESI (*n* = 5)	*N* (%)	LOS (min)	ADM	ADM *p* < 0.001, *η*^2^ = 0.035**	Non-ADM	Non-ADM *p* < 0.001, *η*^2^ = 0.104***	ADM vs. non-ADM
		M ± SD (95% CI)	%	M ± SD (95% CI)	%	M ± SD (95% CI)	*p* (*r*)
Red	1454 (1.55%)	168.8 ± 182.3 (159.4–178.2)	85.4	169.1 ± 183.6 (158.9–179.4)	10.1	162.1 ± 175.6 (133.5–190.8)	0.423 (0.02*)
Orange	17,264 (18.5%)	247.0 ± 177.8 (244.3–249.6)	63.0	250.7 ± 181.8 (247.3–254.1)	28.1	242.9 ± 160.7 (238.4–247.5)	0.854 (0.001*)
Yellow	43,236 (46.2%)	251.3 ± 179.6 (249.6–253.0)	45.4	287.6 ± 208.9 (284.6–290.5)	50.5	219.8 ± 139.6 (217.9–221.7)	< 0.001 (0.190**)
Green	29,807 (31.9%)	162.2 ± 120.9 (160.8–163.6)	10.4	247.4 ± 174.4 (241.3–253.6)	87.1	152.1 ± 107.8 (150.8–153.4)	< 0.001 (0.215**)
Blue	1809 (1.9%)	144.5 ± 140.1 (138.0–150.9)	9.7	246.3 ± 201.2 (216.3–276.2)	87.3	130.5 ± 119.0 (124.7–136.4)	< 0.001 (0.193**)

ADM, admitted; Non-ADM, non-admitted; M, mean; SD, standard deviation; 95% CI, 95% confidence interval of mean; LOS, length of stay; ED, emergency department; MTS, Manchester Triage System; ESI, Emergency Severity Index

*No effect, **small effect, ***moderate effect

In EDs using the ESI triage system (*n* = 5; LOS, 3 h 38 min (95% CI 3 h 17 min–3 h 19 min)), most patients were classified as level 3 (46.2%) and level 4 (31.9%). Patients in level 5 had the lowest LOS of 2 h 24 min (95% CI 2 h 18 min–2 h 31 min), and patients in level 3 had the longest LOS of 4 h 11 min (95% CI 4 h 9 min–4 h 13 min). Overall, the LOS, except for level 1, was > 4 h. No differences in LOS were detected between levels 2, 4, and 5. Notably, the proportion of admitted patients dropped sharply by 75% from level 1 to level 5. From levels 2–5, the proportion of non-admitted patients increased and, correspondingly, the LOS decreased up to 1 h 53 min. No significant differences in LOS were detected for non-admitted patients between triage level 1 vs. level 4/5 as well as for both admitted and non-admitted patients between triage levels 1 and 2 (Table 4, Fig. 2).

### LOS in relation to weekdays and time of year

In the AKTIN ED cohort, the shortest LOS was observed on weekends (3 h 13 min, 95% CI 3 h 12 min–3 h 14 min) and longest LOS on Mondays (3 h 40 min, 95% CI 3 h 39 min–3 h 42 min). Regardless of the day of the week, longer LOS was observed for admitted patients than for non-admitted patients with a range of 58–70 min. Relevant seasonal fluctuations were not observed.

### LOS depending on emergency care levels

For comparison of LOS in terms of the emergency care levels, one ED classified as a basic emergency care provider was excluded from the calculation to preserve the data protection concept of the AKTIN registry. EDs that are classified as extended emergency care providers have 31 min shorter LOS than clinics that are classified as comprehensive emergency care providers (3 h 10 min vs. 3 h 41 min, *p* < 0.001). Additionally, the LOS was higher for admitted (EECP 3 h 48 min vs. CECP 4 h 26 min, *p* < 0.001) patients than for non-admitted patients (EECP 2 h 45 min vs. CECP 3 h 19 min, *p* < 0.001). However, both EECP and CECP showed a difference in LOS between admitted vs. non-admitted patients at 30 and 40 min, respectively. For trauma patients, no difference between the emergency care levels was found. However, non-trauma patients treated by CECP had 36 min longer LOS than those treated by EECP (*p* < 0.001). With EECP, patients with abdominal pain and shortness of breath had an LOS of 3 h 38 min and 3 h 37 min, respectively. With CECP, the LOS was 47 min (abdominal pain) and 64 min (shortness of breath) higher than with EECP.

## Discussion

Our results revealed important findings for the development of strategies to improve ED performance. The mean LOS of 3 h 28 min for all patients included in the study is higher than that reported in other studies. Rygiel et al. [19] reported an LOS of < 3 h, and Biber et al. [20] described a median LOS of 1 h 47 min with a maximum LOS of > 3 h. In contrast to our analysis, both studies were single-centre analyses, including only 29,391 and 4653 patients, respectively.

With respect to patient-related factors, gender differences between the LOS in EDs were not detected in our study. In line with the findings of other studies [20, 36], our results show that the LOS and the percentage of admitted patients increase with age. Self-admitted patients had a shorter LOS of up to 60 min than patients with other modes of referral. However, the rate of admittance for inpatient treatment of self-admitted ED patients was 35% below that for patients referred to the ED by a general practitioner.

Regarding the leading symptoms and disease-associated factors, patients with cardiac chest pain, shortness of breath, or abdominal pain had the highest LOS in the ED. For all three main symptoms, a differential diagnosis must rule out a serious, potentially life-threatening condition, such as acute myocardial infarction or acute abdominal pain. Sometimes waiting for lab results or monitoring the patient in the initial phase is needed. This may also explain the non-existent differences between inpatient and outpatient treatment in some of these groups. Non-admitted trauma patients were discharged from the ED relatively quickly at 2 h 15 min compared with their admitted counterparts at 3 h 40 min (*p* < 0.001). In our study, the shortest LOS was found for the leading symptom "laceration/puncture".

For acuity assessment, our study showed a comparable association of the LOS with the triage level, which was already described in previous publications [19, 37]. Thus, the LOS was up to 2 h shorter for patients who required immediate or very urgent care (levels 1 and 2) and those who were admitted to the hospital after ED treatment than for patients in levels 3–5.

In our study, hospital admission of emergency patients was found to be one of the major relevant factors associated with a higher LOS. The longer LOS of admitted patients compared with non-admitted patients are attributable to the amount of time spent in the ED for necessary diagnostics. The waiting time for patients to be transferred to the specialist department plays a major role because the bed capacities in specialist departments are usually not available ad hoc due to the so-called access block [14]. Only in two scenarios, hospital admission was not associated with a higher LOS. In EDs using MTS as a triage system, admitted patients classified in the categories of red and orange had 33-min and 56-min shorter LOSs, respectively. In the second scenario, admitted patients with extremity weakness/symptoms of cerebrovascular accident as presenting complaint also had up to 39 min shorter LOS than non-admitted patients with the same presentation complaint. These are patients with suspected stroke needing immediate transfer to a special monitoring unit (stroke unit) according to international standardised ED protocols.

For organizational factors, our study results revealed significantly shorter LOS on weekends than on working days. The LOS was surprisingly up to 40 min shorter, but the number of patients was only 2% lower on average. Considering that the number of staff is usually reduced on weekends, this finding is remarkable. A possible explanation for this observation could be the increased use of the EDs by patients with minor complaints and less diagnostic effort outside the opening hours of general practitioners, but also the absence of referrals of more severe cases by general practitioners on weekends. Another explanation could be that on weekends, patient care is sometimes more focused as emergencies do not have to be scheduled between elective cases.

Several countries introduced "time targets" to evaluate emergency medical services, enabling a comparison of our findings with those in the international context. The 4-h target implemented in Australia (80%), Great Britain (98%), and Canada (90%) was not achieved in our cohort of AKTIN EDs during the observation period: 69.6% of patients were discharged from the ED within 4 h, 88.2% after 6 h, and 5% of all emergency patients left the ED after 8 h. In comparison to international findings, our findings could be explained by differences in general and structural settings within the EDs. Particularly, EDs in Anglo-Saxon countries have been independent specialist departments for many years, and clinical emergency medicine represents a clearly defined medical specialty. In contrast, the minimum structural and procedural requirements as well as the establishment of a central ED were not legally binding in Germany until 2018 [25]. Baier et al. describe the different structures of emergency departments from different countries [38].

The reasons for the longer LOS in EDs with a higher care level may be due to different patient characteristics and/or the availability of more diagnostic and therapeutic options. However, a longer LOS in the ED does not inevitably result in a poorer quality of care: the discharge or transfer of an emergency patient should only be initiated after the completion of comprehensive diagnostics to ensure patient safety and to reduce the risk of adverse events even if it involves a longer LOS. Therefore, we suggest classifying the LOS as a performance measure rather than a quality indicator [39]. The influence of LOS on the medium-term patient outcome and thus its value as a quality indicator is currently being examined in the ENQuIRE project by linking AKTIN registry data with claims data from one public health insurance [40].

### Limitations

Our data is process-generated data. Hence, the validity of documented timestamps must be considered when evaluating LOS. While the time of admission is usually recorded very accurately (entering insurance information into the system and performing the triage), the validity of other timestamps remains a challenge in most EDs: When or how is the "medical contact" operationalized or the "discharge" scheduled? For example, a bias can arise if patients leaving the ED are not "discharged" immediately from the documentation system but only later, for example, by administrative staff closing the patient file. This can particularly occur during resuscitation, where emergency treatment is in focus, and documentation is performed with a time delay.

### Conclusions

Our study shows that the total LOS in the ED mainly depends on patient-related factors (age), disease-related factors (presenting complaint and triage level), and organizational factors (weekday and disposition). Analyzing large datasets of the AKTIN registry allows for a valid evaluation of the LOS in EDs at all emergency care levels. Even if many of the influencing factors, e.g. age, gender and presenting complaints, cannot be changed as such, their identification is important. Real-time tracking of data in the ED as well as an optimized management of processes may help to avoid delays in diagnostics and interventions and improve the flow of patients through the ED. In addition, a baseline for external comparisons is needed. Our results support the development of strategies to optimize the processes in the ED. This applies, for example, to the reduction of the “boarding time” between treatment completion and discharge time. Future studies should therefore not only look at the LOS as a whole, but also take a closer look at such sub-processes during emergency treatment.
